# Supervised and Self-Supervised Learning for Assembly Line Action Recognition

**DOI:** 10.3390/jimaging11010017

**Published:** 2025-01-10

**Authors:** Christopher Indris, Fady Ibrahim, Hatem Ibrahem, Götz Bramesfeld, Jie Huo, Hafiz Mughees Ahmad, Syed Khizer Hayat, Guanghui Wang

**Affiliations:** 1 Department of Computer Science, Toronto Metropolitan University, Toronto, ON M5B 2K3, Canada; 2 Department of Aerospace Engineering, Toronto Metropolitan University, Toronto, ON M5B 2K3, Canada; 3 IFIVEO Canada Inc., Windsor, ON N8W 0A6, Canada

**Keywords:** computer vision, action recognition, temporal action localization, semi-supervised learning, supervised learning, real-time feature extraction, assembly line monitoring

## Abstract

The safety and efficiency of assembly lines are critical to manufacturing, but human supervisors cannot oversee all activities simultaneously. This study addresses this challenge by performing a comparative study to construct an initial real-time, semi-supervised temporal action recognition setup for monitoring worker actions on assembly lines. Various feature extractors and localization models were benchmarked using a new assembly dataset, with the I3D model achieving an average mAP@IoU=0.1:0.7 of 85% without optical flow or fine-tuning. The comparative study was extended to self-supervised learning via a modified SPOT model, which achieved a mAP@IoU=0.1:0.7 of 65% with just 10% of the data labeled using extractor architectures from the fully-supervised portion. Milestones include high scores for both fully and semi-supervised learning on this dataset and improved SPOT performance on ANet1.3. This study identified the particularities of the problem, which were leveraged and referenced to explain the results observed in semi-supervised scenarios. The findings highlight the potential for developing a scalable solution in the future, providing labour efficiency and safety compliance for manufacturers.

## 1. Introduction

Deep learning techniques have made tremendous advancements in the past decade and have been successfully applied to many practical problems, such as object detection [1,2], segmentation [3,4], depth estimation [5], and system prediction [6,7]. Modern factory work is becoming increasingly complex, often lacks adequate supervision, and requires human cognition and dexterity [8]. As a result, deep learning techniques are desired for monitoring human activity in industrial settings to ensure safe, quality work [9]. Some automated factory supervision methods employ a variety of sensors, which may be worn by the worker or placed on the tools [10,11]. Additional sensors would provide more information but have additional monetary costs, which could dangerously interfere with the worker or may require difficult-to-synchronize sensor fusion [12]. Günther et al. [11] employs tool-only sensors, forgoing cameras in favor of privacy and simplicity, but the authors acknowledge their limited effectiveness. Both ML and DL classifiers have disadvantages; the former requires features, and the latter requires more data.

Several works approach action seeking by viewing actions as a combination of axiomatic base actions, be these therbligs [8], MTM-1 [13], or tool movements along the cardinal rotational axes [11]. Though atomic actions are interpretable and feature data-efficient, hand-designed components, they may lack adaptability as arbitrary actions and may not be easily separable in terms of basis. Therefore, decomposition based on confusion matrices of [8] may not be effective in all cases. As factory worker tasks are numerous and ever-changing, hand-designed components may not be ideal for future scalability despite their short-term data efficiency. Utilizing pose-estimation skeletons is an alternative, be it full-body [8,14] or hands [13]. Both decompose an action into related moving parts or skeletons for data efficiency and explainability. However, purely skeleton-based methods may ignore the surrounding context (including the tools in the worker’s hands), which is exacerbated if only the hands are considered. A CNN solution may also be simpler.

A seminal conclusion of the deep learning era is that the neural network’s nature as an automatic universal function approximator is, provided enough data, more effective than hand-designed components [15]. General-use models should be considered as candidates for our niche application due to the vast body of research conducted on them. Leveraging the data efficiency of base actions and skeletons while still using a standard architecture is possible through self-supervised learning. Real-time temporal action localization (TAL) classifies and localizes actions as they occur, making it appropriate for detecting and classifying actions in factory settings. Generally, an extractor encodes video clips into feature vectors and then uses a head to classify that clip and predict the start and end times of the action to which it belongs [16].

To formalize TAL, an input video of *N* frames is represented as tensor $X_{v}\in R^{3\times H\times W\times N}$. To be manageable for extractors trained for trimmed video classification and with finite temporal context, $X_{v}$ is divided into clips. A clip $X_{v,i}\in R^{3\times H\times W\times S_{stack}}$ is a contiguous subset of the video frames of length $S_{stack}$ (the stack size) that starts at frame $i\times S_{step}$ (the step size). TAL starts with a feature extractor ϕ, a function (often a CNN) trained to map the clip to a feature vector $\phi \left(X_{v,i}\right)\in R^{D}$, and a far more informationally dense tensor. *D* is the feature dimension, which is the number of features that the architecture can send downstream. Assuming the video was divided into *T* clips (can be viewed as a “temporal location”), the extracted feature map of the video is $\phi \left(X_{v}\right)=R^{T\times D}$. The “head” will, from this feature map, make the prediction expected in TAL. Namely, this is a set $\hat{Y}=\left\{\left({\hat{y}}_{i,s},{\hat{y}}_{i,e},{\hat{y}}_{i,c}\right),\ldots \right\}$ of actions, which is identified by the start ${\hat{y}}_{i,s}$ and end ${\hat{y}}_{i,e}$ time of the video where the action occurs and by the class ${\hat{y}}_{i,c}$ of the action. This relates directly to the goal of TAL to find when actions occur and the types of those actions. For semi-supervised purposes, a model pretrained on a non-TAL task can be trained using this TAL paradigm.

We assembled a study based on recent models fitting our criteria, which is defined as follows. When recent temporal action localization systems achieve their impressive performances [17,18], their best results typically leverage features extracted using large models [19,20], which may not provide the real-time performance needed for correcting worker actions. I3D [21] and R(2+1)D [22] are extractors based on successful CNN image classifiers with kernels extended to the temporal dimension; the former simply converts all 2D filters to 3D, and the latter follows each 2D kernel with a separate 1D to respect the differing natures of space and time. We also seek methods that reduce the workload for annotators, which are presented in Table 1. Self-supervised methods use these extractors but also include a pretraining regimen based on calculable pretext tasks to promote dataset understanding [23,24,25]. SSP [23] keeps training on fewer labels and is consistent by using an exponential moving average of the weights. SSTAP [24] adds a branch tackling clip shuffling to better understand feature order. SPOT [25] parallelizes classification and localization and refines action/background borders with a contrastive loss. Though SPOT has been effective, it could be improved through adaptations based on assumptions from the dataset. Accurate localization in TAL is challenging, as adjacent video frames are nearly identical. We incorporate a novel loss to produce firmer action/background boundaries by contrastively repelling the action and background predictions. A focus of previous TAL solutions has been to find relevant pretext tasks, though these will inevitably have a distribution that differs from the downstream task. We intersperse training epochs within the pretraining to direct the losses toward the downstream task. Doing this will reduce the gap that the limited quantity of supervised data would need to correct for.

Within the broader context of action recognition research, we study real-time, supervised, and semi-supervised temporal action localization solutions. Previous works have considered non-real-time segmentation models [27], considered factors affecting TAL in non-factory settings [28], inspected repetitive actions for time estimation [29], looked at human-centric assembly [30], and have applied complex mesh-based systems, but none have addressed our particular niche. Using a new factory dataset named Undercover Actions (UAs) as a benchmark, each pipeline’s applicability to the assembly action recognition task is assessed. UAs is not publicly available at the time of publication to preserve the privacy of workers, but a generalizable analysis is provided.

This paper makes the following contributions:

1. A comparative study on a new assembly line action recognition dataset with the intention of comparing real-time and/or self-supervised solutions. Analysis of the dataset is provided to direct further generalization of the understanding of temporal action localization in factory settings.
2. Modifications to SPOT, an existing semi-supervised model, to enhance its performance on ANet1.3 [31] and provide good results on the assembly line action recognition dataset.

## 2. Materials and Methods

### 2.1. Undercover Assembly (UA) Dataset

**Figure 1:**
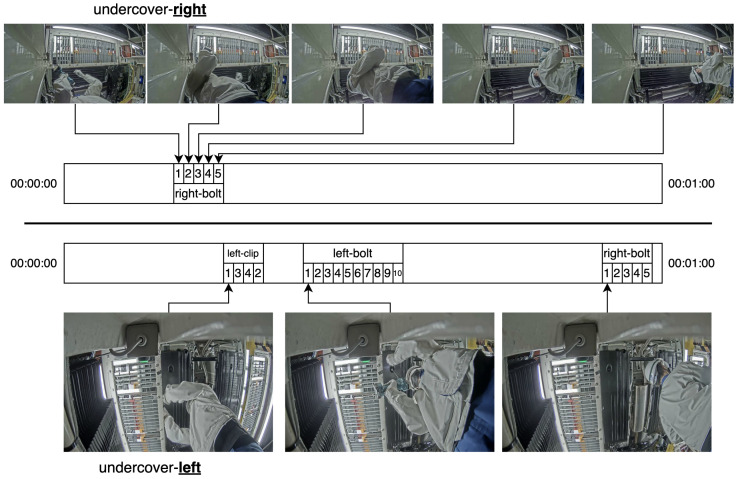
Qualitative analysis of UA’s nature, represented as annotated timelines of two select videos (selected as both are 1 min in length and have an annotation for each class visible to their side). For example, the image frame on the bottom left is a frame from an annotation of class left-clip-1, which tends to occur early in videos. Right-camera (undercover-right) videos have five classes, all right-bolt. Left-camera (undercover-left) videos have classes from left-clip (visible hands, no bolt tightener), left-bolt (visible hands, bolt tightener), and right-bolt (but from this camera, the right-bolt hands are obstructed).

This study focuses on a typical assembly-line application, Undercover Actions (UAs), as shown in Figure 1. UAs is a collection of untrimmed videos from a pair of cameras placed under (hence the “Undercover” part) one stage along a vehicle assembly line in which a plate is attached to the undercarriage. Each video contains a single round of actions on a single vehicle. The 995 videos (with a mean duration of 71 s) are split into five equal folds for training/testing. There are 19 action classes pertaining to the bolts and clips being installed; from the right camera, which contributes 45% of the videos, only 5 classes are visible. The classes are left-bolt-*X*, X∈{1,…,10}, right-bolt-*X*, X∈{1,…,5}—that represent the installation of bolts on the left and right side of the plate—and left-clip-*X*, X∈{1,…,4} to represent the installation of the clips on the left side of the plate. The factory footage contains sensitive information, including faces, logos, and techniques and is therefore not currently publicly available.

**Figure 2:**
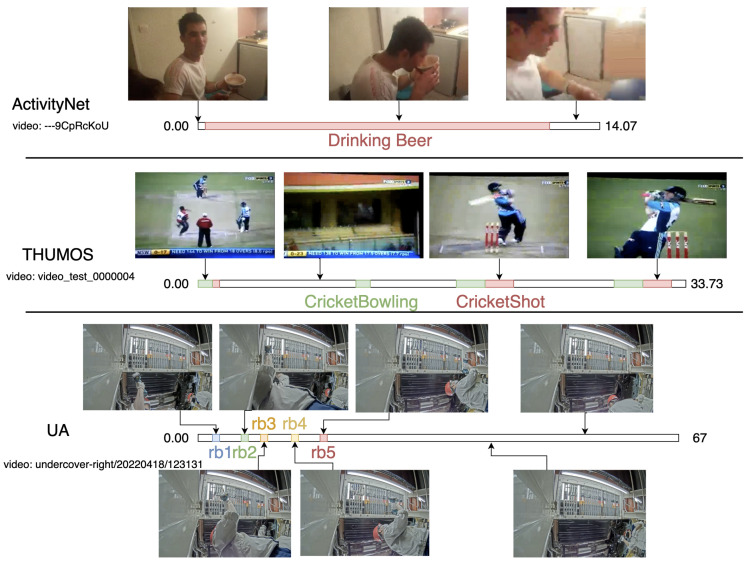
A spatiotemporal comparison of one select video from each dataset used in this paper. Timelines are shown with end times (duration, in seconds). Frames from along the annotated timeline are presented.

Compared to standard TAL benchmarks THUMOS’14 [32] and ANet1.3 [31], most UA videos include one annotation for every class in a constant order across the videos. Instances are very short compared to those of other benchmarks (visualized in Figure 2), as they pertain to a particular bolt or clip being attached. The classes are spatially separated when done correctly, suggesting that location could be useful for verifying identity/correctness. Depending on the view, this indicates that large spatial regions may exist where no correct actions could occur. It can be reasonably assumed that general factory work would also be ordered and repetitive, with short actions corresponding to the attachment of some component to the product at a particular station. UAs represents a useful starting use case that should be generalized, as many products are manufactured on auto-like assembly lines.

Table 2 shows the statistics of UAs compared with large standard TAL benchmarks. The higher mAP suggests that TAL on UAs is an easier problem than on THUMOS/ANet1.3. The UA backgrounds are mostly static, making them easily ignored by attention-based extractors [19], but provide no context. Assuming the extractor is trained on UAs, the spatial location of the classes would become a useful feature; even without training, the spatial locations would still affect the feature for a downstream head to decipher.

### 2.2. Models

**Figure 3:**
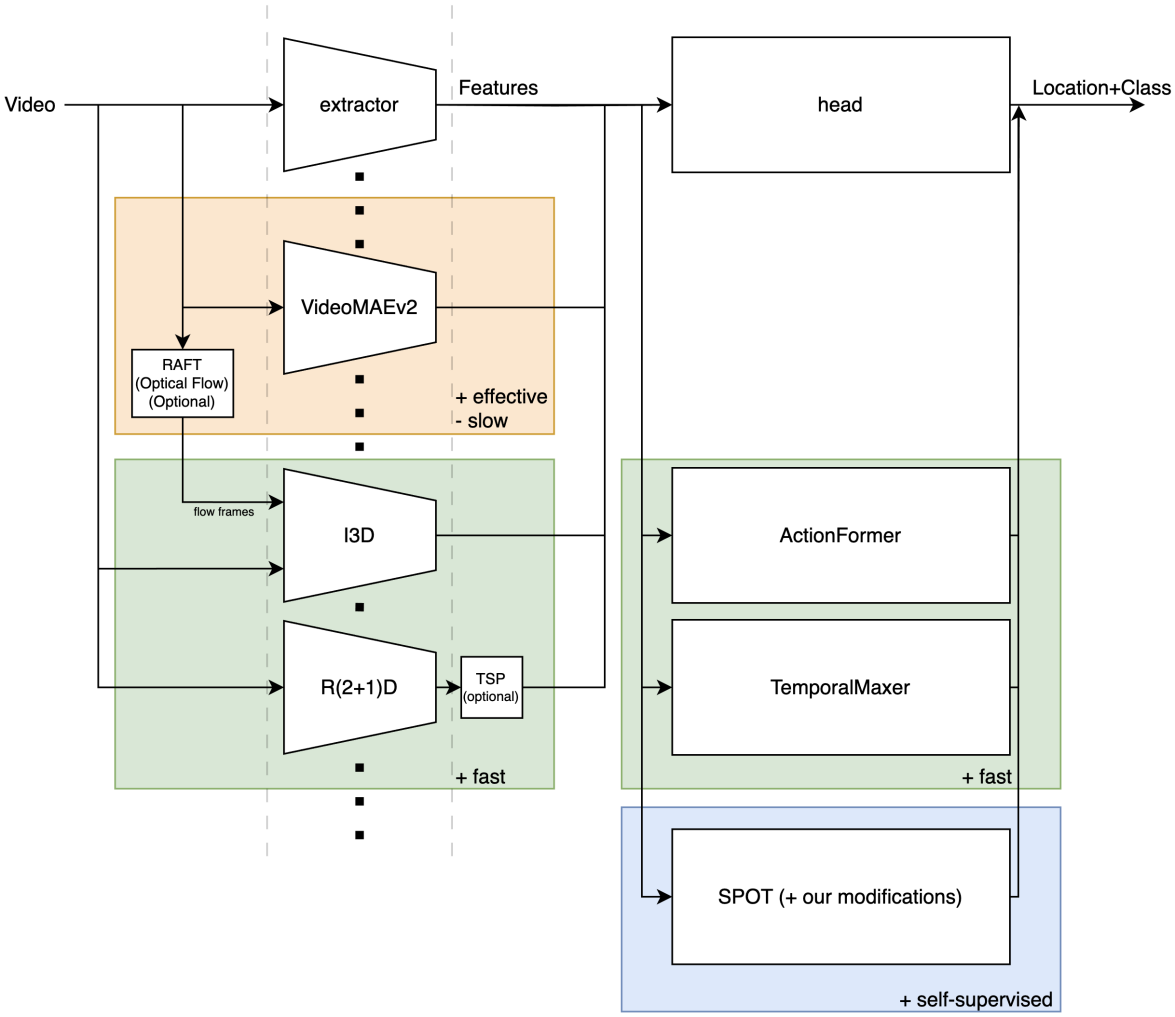
A summary of the extractors and localizers used in the comparative study and their relationships. Each complete path represents an architectural base for experiments.

In this study, we selected four backbones (VideoMAEv2 [19], I3D [21], R(2+1)D [22], TSP [33]), RAFT [34] for optical flow generation, two supervised heads (ActionFormer [35], TemporalMaxer [18]) and one self-supervising head (SPOT [25]). Several rationales governed this selection. For a rich comparative study, we sought a group of SOTA TAL components with diverse architectures. However, since real-time performance is critical, we strongly prioritized simple, real-time models. Similarly, we hope to deploy these models in a variety of factories, so easy-to-generalize convolutional systems are preferred. I3D and R(2+1)D were reasonable choices, as they are based on tried-and-true Inception and ResNet modules. We also sought to observe the effect of the dataset’s nature. For understanding repetitive sequences, TSP was selected for its consideration of global information and background clips, and was chosen VideoMAEv2 for its attention. For short actions, TSP was tuned for temporal sensitivity. These are summarized in Figure 3. Among the backbones, these are the following descriptions: VideoMAEv2 is a large attention-based model, which is not real-time but has been selected to inspect how well an automated system can perform on this dataset; I3D and R(2+1)D are real-time, fully-convolutional models structured as 3D Inception and (2+1)D ResNet, respectively; RAFT was used to evaluate I3D in a two-stream setup; TSP specializes R(2+1)D (among others) for TAL by explicitly predicting binary action/background for sharper temporal boundaries. ActionFormer and TemporalMaxer are both real-time SOTA heads that map the extracted features to the set of starttime-endtime-class triplets expected by TAL. Both use a pyramid of blocks to collect features at various scales, but ActionFormer uses vanilla transformer blocks (with downsampling to form the pyramid) to perform attention, whereas TemporalMaxer performs a simple max-pooling operation for its selection of critical features. SPOT is another classifier/localizer head that is a top-performing system for semi-supervised tasks through random start–end generation (a TAL-oriented preliminary task) and a contrastive boundary refinement. This study therefore provides comparisons across extraction speeds, supervision proportions, and architectures.

#### 2.2.1. Supervised Models

The supervised portion of the comparative study sought to achieve good results on UAs and indicate an extractor capable of informative features. The billion-parameter, top-performing masked autoencoder VideoMAEv2 [19] is not real-time but serves as a high watermark for other extractors and could adopt continual learning roles. VideoMAEv2 is attractive as a large-scale model for comparison, since its dual-masking strategy provides efficiency to counteract its size. The two base architectures recruited as real-time extractors are I3D [21] and R(2+1)D [22], which were chosen due to their speed as effective fully-convolutional extensions of image models.

I3D [21] is Inception [36] (to ensure that multiple scales are represented), where each $k^{2}$ kernel becomes a $k^{3}$ kernel pretrained on ImageNet [37]. The network-in-network is useful during the “still video” phase, as it forces the model to look temporally at different scales and for maintaining efficiency with a higher weight count. However, the spatial and temporal dimensions have different distributions, and the 3D kernel dramatically increases model size. I3D can be used as a two-stream model; an identical I3D architecture can learn effective optical flow features given optical flow frames generated from RGB frames using a model such as (used in this paper) RAFT [34]. Additional features increase the average precision but slow the inference to below a real-time rate.

R(2+1)D [22] is a ResNet [38] variant for videos that follows each original 2D convolution for the spatial (image) dimension with a 1D convolution in the temporal dimension. Consecutive layers (a group of residual blocks) output feature maps of decreasing size in accordance with the standard pattern of encoding and scale hierarchy and maintain the benefits of ResNet for the vanishing gradient problem. R(2+1)D was used here with and without Temporally Sensitive Pretraining (TSP) [33]. TSP counters the action/background imbalance problem and enhances localization through a branch to explicitly discriminate between action and background.

Two fully supervised heads were selected to map the features to a set of predicted annotations: ActionFormer [35] and TemporalMaxer [18]. Both use a pyramid structure to generate and aggregate features at multiple scales and use lightweight decoders to provide predictions at temporal locations. ActionFormer uses transformer blocks and downsampling to form the pyramid, constituting a powerful yet still real-time method. TemporalMaxer’s pyramid tiers are composed of two 1D convolution layers followed by a max-pooling layer. This results in a simple, lightweight system. The convolution layers in the encoder do not reduce the dimension of the features, mixing the encoder’s features to increase their applicability to the current task. By sending all features through max-pooling layers, the model’s decision is based on which responses are strongest. These responses come from weights learned to provide features that measure their relevance to the class of the given case. The responses should be strongly class-discriminative through this filtering, although features of secondary importance would be lost.

#### 2.2.2. Semi-Supervised Model and Augmentations

The standard semi-supervised TAL pipeline follows the extractor with a localizer, which produces a set of proposals for the locations of general actions [23,24] to be followed with a classifier such as UntrimmedNet [39]. This separation simplifies the problem in the face of the challenge of limited data, yet this causes errors to be propagated forward. The most recent SPOT model [25] employs a lightweight transformer to add temporal context to the input features, produces the localization and classification predictions in parallel, and then joins these branches using contrastive learning to enforce action/background boundaries. Due to this structure, SPOT is faster and better performing than its predecessor SSTAP [24].

Our SPOT pipeline has been mostly unchanged from the original. We applied the following series of augmentations to increase SPOT’s performance on ANet1.3 and UA.

**A. Pretraining-Training Alternation.** The general SSL pipeline has a first stage, which pretrains a deep architecture on a pretext task that is trivially decidable, followed by a second stage of supervised fine-tuning on the labeled dataset portion using layers that have gained insight into the dataset distribution but are still relevant to the task [40,41]. However, the discrepancy between tasks may result in suboptimal features [42]. The recent BiSSL [43] jointly optimizes pretext and downstream via framing as a bilevel optimization problem, though the solution does incur significant additional computational complexity. We intersperse some training epochs into the pretraining phase to regulate and direct the pretraining toward the downstream task. RIFLE [44] also performs repeated training but re-initializes fully connected layers randomly, which may lead to instability. As per Algorithm 1, pretraining and training epochs were alternated to direct the weights toward the optimal for the downstream task significantly earlier. As determined empirically, 30 epochs of pretraining and 40 epochs of training, alternating every 3 epochs, was found to be effective. Multiple epochs at a time ensure that sufficient progress is made for directed training. More pretraining than training ensures that the final weights are directed at the training task, though excessive training epochs would naturally lead to overfitting through a lack of data and forgetfulness regarding the pretraining task.
**Algorithm 1** Pretraining–training alternation**Require:** SPOT model $f_{\theta }$ with parameters θ**Require:** features ${\left\{x_{i}\right\}}_{i=1}^{N}$**Require:** Training dataset $D_{train}={\left\{\left(x_{i},y_{i}\right)\right\}}_{i=1}^{N}$**Require:** Pretraining dataset $D_{pretrain}={\left\{\left(x_{i},y_{i}^{\prime }\right)\right\}}_{i=1}^{N}$**Require:** loss functions $L_{pretrain},L_{train}$**Require:** epoch schedule $e_{schedule}=rounds*\left(blocksize*\left[e_{pretrain}\right]+blocksize_{train}*\left[e_{train}\right]\right)$    ▷ List concatenation; example $e_{schedule}=2*\left(1*\left[e_{p}\right]+2*\left[e_{t}\right]\right)=\left[e_{p},e_{t},e_{t},e_{p},e_{t},e_{t}\right]$**Require:** learning rate η**Require:** number of epochs *E***Require:** batch size *B***Ensure:** Trained model parameters θ  1:Initialize model parameters θ (e.g., randomly or with a predefined strategy)  2:**for** epoch in $e_{schedule}$ **do**  3:      **if** epoch = $e_{pretrain}$ **then**  4:            let: $D=D_{pretrain}$  5:            let: $L=L_{pretrain}$  6:            let: $y=y^{\prime }$         ▷ For brevity while discussing the target value  7:      **else if** epoch = $e_{train}$ **then**  8:            let: $D=D_{train}$  9:            let: $L=L_{train}$10:      **end if**11:      Shuffle the training dataset *D*12:      **for** each batch $B_{j}\subset D$ **do**13:            Compute predictions: ${\hat{y}}_{i}=f_{\theta }\left(x_{i}\right)\;\forall \left(x_{i},y_{i}\right)\in B_{j}$14:            Compute loss: $L_{batch}=\frac{1}{|B_{j}|}\sum_{\left(x_{i},y_{i}\right)\in B_{j}}L\left({\hat{y}}_{i},y_{i}\right)$15:            Compute gradients: $\nabla_{\theta }L_{batch}$16:            Update parameters: $\theta \leftarrow \theta -\eta \nabla_{\theta }L_{batch}$17:      **end for**18:**end for**

**B. Reconstruction Loss and Temporal Crop.** An MSE reconstruction loss was applied to pairs of input and SPOT encoder features, before and after the temporal crop, to ensure robustness. Though UAs’ classes might tend to appear at particular parts of a video, this is not guaranteed nor will this be guaranteed at deployment.

**C. NLLLoss for Classification.** The NLLLoss [45] replaces the MSELoss for the classification branch as a more appropriate loss for classification and to better encourage high-confidence predictions. If this is preceded by a softmax, NLLLoss receives values that are not only restricted to the range [0,1] for easy computation, but they are probabilities which sum to 1, which NLLLoss can process as a multinomial MLE. The negative log function will encourage high confidence by severely punishing low confidence scores.

**D. MSE+Contrast for Localization.** During pretraining, the MSELoss was found to exceed the performance of a BCE/Dice combination, since the former produces smooth gradients for generalizability. For the localization branch, we proposed the loss in Equation (1) to push action and background predictions apart for sharper temporal boundaries. In addition to the binary cross-entropy (BCE) loss, a variance term (variance of the predicted mask given as σ(x)) was added to resist small prediction value ranges, subtracted from 0.25. This is because the maximum possible value of the variance of the mask is 0.25, as proven in Appendix A. The ${\bar{x}}_{action}$ and ${\bar{x}}_{background}$ (a justification for this selection is given in Appendix B) refer to the means of the scores on ground truth actions and ground truth backgrounds, respectively, and we pushed these apart. To address foreground/background imbalance, the action term was scaled by a coefficient (we set *w* = 5 in our experiment as determined empirically). Divide-by-zero errors were avoided by adding 1 to the mean denominator (ground truth action count).

(1) $$L_{mask}=BCE\left(x,y\right)+\left(0.25-\sigma \left(x\right)\right)+w\left(1-{\bar{x}}_{action}\right)+{\bar{x}}_{background}$$

**E. Reduced Temporal Scale.** ANet1.3’s videos generally have a single long action, approximating a classification problem (as shown in Table 2), and hence, a smaller scale is advantageous, since a naive localizer, which uses the entire video as an action prediction, may do very well on these videos. However, UAs’ actions are short, and therefore, the temporal scale must be increased markedly to avoid the harsh penalty of failing to recall the actions.

## 3. Experiments and Results

This section presents the comparative study. The extractors—the first component of the TAL pipeline applied to supervised and self-supervised—are inspected in Section 3.1.1. The objective is to determine how the key hyperparameters of architecture, step size, and stack size relate to runtime and mAP. The heads—the second component—are compared in Section 3.1.3 to ensure real-time performance and compare the mAP for a given set of features. The highest-mAP experiment for extractor–head combinations is given in Section 3.3. To showcase the dataset’s properties and to direct future work, Section 3.1.2 analyzes the effect of cropping out the static background. Semi-supervised work is discussed in Section 3.2.

We implemented all learning models and compared their performance. The implementation setups and the hyperparameter settings for the ActionFormer and TemporalMaxer heads are given in Appendix C. Within the Appendix, Table A1 summarizes the software environment and the code bases used for the models in the comparative study. Table A2 summarizes the hyperparameters used by the supervised heads. The hyperparameters shown in Table A2 were fixed for all experiments using the supervised heads; only the heads’s stack and step size varied, set equal to the clip size for each experiment in Table 3 to ensure alignment between features and heads.

Unless otherwise stated, all experiments were carried out by sending the entire dataset through a non-fine-tuned extractor, and then the features were passed into the supervised or self-supervised head. The inference time for RGB and flow features assumes a combined algorithm, though flow frames require prior computation with separate weights, making total times comparable. All experiments were conducted with the same training and testing folds; semi-supervised experiments utilized a portion of the same training folds and used systematic sampling based on the necessary sampling interval.

### 3.1. Supervised Experiments

This subsection covers the experiments using a supervised training/evaluation scheme and supervised heads, i.e., ActionFormer or TemporalMaxer.

#### 3.1.1. Extractor Comparison

Extractors are critical, as they must effectively filter superfluous pixels and include features relevant to the task and are required by all the supervised and self-supervised heads examined here. These extractors are evaluated for their feature quality (as measured by ActionFormer’s mAP) and if they are real-time (defined as the FPS of the system exceeding the FPS of the video). Some hyperparameters/factors can be intuitively identified as having an outsized effect on the runtime and mAP. One natural one is the model, which will incorporate not only the architectural achievements of those models but also the parameter count. The clip size is the other key hyperparameter for runtime and mAP, since it directly controls how much information is provided (and must be processed); step_size = 4, num_frames = 16 indicates a clip of 16 frames is used, where the starting frames of adjacent clips are four frames apart. The case where step_size = num_frames would create a non-overlapping partition where, except for the incomplete remainder frame at the end, each frame is included in exactly one frame. For each row, ActionFormer’s clip size setting was matched to the clip size of the extractor; this provided compatibility to maximize mAP, and ActionFormer’s setting was insignificant for runtime in comparison to the extractor.

Table 3 compares the extraction time and mAP based on various extractors on UAs. One conclusion is that non-real-time solutions do not provide a justifiable gain in mAP for the loss of real-timeness. The small VideoMAEv2 configuration, VideoMAEv2-small, did not provide significantly better results than its real-time counterparts. It did provide better results considering the larger step size, which would make each frame appear in only one clip, but increasing the step size would make VideoMAEv2-small even further from the real-time requirement. VideoMAEv2-small has a similar parameter count to the real-time solutions; differences in runtime and performance can be explained by its attention module. In comparison to an RGB-only I3D, the two-stream variant took 3× as long to run yet provided a less than 1% mAP boost. The requirement that RAFT computes a H×W×2 flow frame for each H×W×3 frame used prior to the extractor run explains the runtime disparity. The minuscule improvement due to flow frames (I3D-164 vs. I3D-R-164 in Table 3) could be explained by the sufficiency of spatial location and the minimal movement associated with each class. Except for a powerful model such as VideoMAEv2, it would appear that step_size <= 8 is needed for good results. UAs’ video frame rates vary but are typically about 20 FPS; therefore, step_size = 16 is nearly a full second gap, which is too much for a dataset in which actions typically last about 1 s. R(2+1)D has more weights (and skip connections) than I3D, and both were pretrained on Kinetics [21], yet I3D performed slightly better; though this dataset’s action annotations are ordered and repetitive, spatial location is more critical for classification, and therefore, R(2+1)D’s 1D temporal convolution may not be providing good value. Despite having fewer trainable parameters, I3D was not faster than R(2+1)D. This may result from the computationally expensive 3D convolutions and the multiple branches associated with the Inception modules. The 85.00% achieved by I3D-164 is the best yet result for a real-time supervised solution on UAs, and so we focused on this extractor configuration for the semi-supervised experiments.

#### 3.1.2. Cropped Experiments

**Figure 4:**
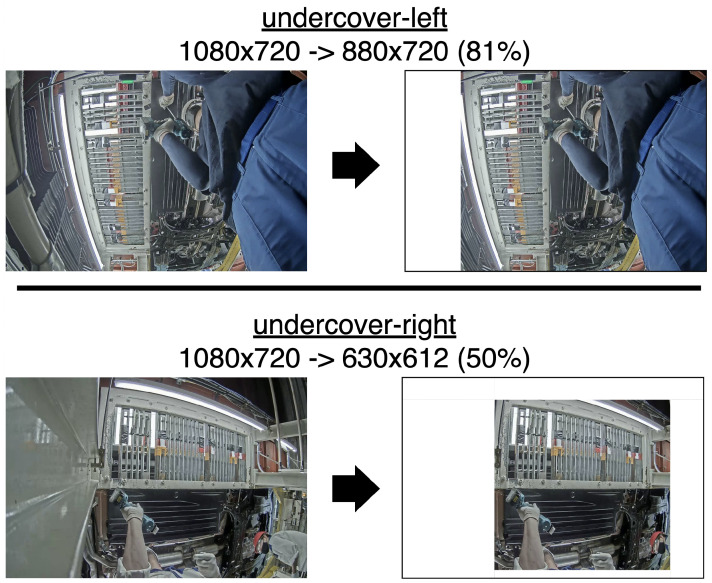
A diagram of the simple crop, showing which subset of the original frame pixels are kept for each frame for each side (undercover-left or undercover-right) of the dataset.

In each frame of the assembly video, large spatial regions exist that contain no actions and are static; their inclusion may therefore be a source of confusion while providing no discriminative information. As determined qualitatively and shown in Figure 4, the right half of the dataset only requires half of the original pixels to spatially show all actions. To evaluate the effect of removing these background regions and determine if a sufficient improvement is noted to suggest pursuing a more sophisticated crop, we applied a simple spatial crop to the dataset and ran experiments on it and the original, *ceteris paribus*.

Experiments were run using both attention-based and attention-free extractors—VideoMAEv2 and R(2+1)D—to test the hypothesis that an attention-based extractor should nearly by definition be more effective at recognizing important portions, and therefore, manually eliminating unused regions was less necessary. A copy of UAs, dubbed UA-simplecrop, was created by cropping each 1080×720 UA video to 880×720 (81% of original size) for undercover-left or to 630×612 (50%) for undercover-right. The simple crop, determined qualitatively, is shown in Figure 4.

These cropped videos were fed into the extractors. All videos were resized to the same size as per the extractor’s requirements, and no more automatic cropping was done to produce the results in Table 4. With a strong attention mechanism already in place, VideoMAEv2 benefited less from the rough crop. This experiment suggests that eliminating the unnecessary portions of the video is beneficial for action recognition, though the performance gains were not significant using the rough crop. A stronger crop (centered around the hands), and perhaps some encoding of the location of that crop, may lead to further gains.

#### 3.1.3. Head Comparison

Two SOTA heads were selected for comparison in this unsupervised portion so that a somewhat complex architecture could be compared to an extremely simple architecture. Multiple architectures were used to extract features in Table 3; we sought to determine if the localizer/classifiers were (with respect to each other) sensitive to the extractors or if one head would perform better than the other on all sets of features. In Table 5, the heads are directly compared; top-performing settings for each extractor were used to generate features, which were then sent into ActionFormer and TemporalMaxer and compared for mAP and runtime (inference). The provided inference time is the time required for the entire test set and therefore implies that both heads are real-time.

The UA results in Table 5 confirm that on I3D and R(2+1)D, TemporalMaxer provided worse results (despite having twice as many training epochs as per Table A2) but was slightly faster at inference. I3D and R(2+1)D provide local spatiotemporal features, with minimal global context; TemporalMaxer can aggregate features to achieve global understanding based on these local understandings but may do so less effectively than ActionFormer’s ViT. The ViT’s sufficiency, and ActionFormer’s focus on local self-attention (rather than the global motion context that can also be garnered from optical flow) may explain why TemporalMaxer sees more relative benefit when moving from a one-stream to a two-stream I3D. On VideoMAEv2, however, TemporalMaxer was the better performer. This may be due to an agreement between the head and extractor on local/global context. VideoMAEv2 is a large transformer model which would discover rich long-term relationships. While ActionFormer might be effective at local attention, TemporalMaxer is a pyramid of max-pools so global patterns (such as the near-constant order of actions in videos in this dataset) could be used to great effect to predict when a class might occur. Given that both are real-time, this project recommends the use of ActionFormer, given the choice of these two. Why TemporalMaxer’s inference for VideoMAEv2 took nearly twice as long as ActionFormer’s is highly unusual and unclear and may have a cause external to the program run.

### 3.2. SPOT

SPOT is used for experimentation as a means of achieving good semi-supervised results on UA. These modifications were successful in improving SPOT’s performance on ANet1.3 beyond the result claimed in the SPOT paper, an additional contribution of this work analyzed in Table 6.

For SPOT experiments, the main environment details are given in Table A1. For extracted features, experiments using ANet1.3 use the I3D features from the SPOT repository [25]. This facilitates a fair comparison between the original SPOT, our implementation and our modifications (Table 6) while using the general I3D architecture from our other experiments. For UA, SPOT experiments use the same single-stream I3D features that provided the 85.00% result in Table 3, since this was the best real-time result. As justification, I3D’s winning performance on supervised ActionFormer evidenced its ability to extract informative features from UA’s clips. Though not all extractors performed better on ActionFormer (Table 5), given SPOT’s attention mechanism it may be more similar to ActionFormer than to TemporalMaxer. I3D was also not fine-tuned on UA to achieve its results, requiring no labelled data percentage assumptions. Table 7 has the best SPOT results achieved on UA; this result was attained using all of the contributions outlined in Table 6, although the temporal scale must be increased rather than reduced to work with the short annotations of UA. The details of the contributions were selected in part based on the regression analysis in Table A3 and the associated data in Appendix D.

Table 6 showcases our evaluation on ANet1.3. The results demonstrate that the pretraining alternation provided a boost of 1% mAP versus the base implementation, suggesting that the early introduction of the downstream task provided a useful direction for the pretraining. Ensuring that reconstruction loss was applied before and after the temporal crop provided another slight boost through additional information. Using NLLLoss was found to be more beneficial, agreeing with the prior that NLLLoss’s use of log-likelihood is more agreeable with classification. A boost of 2% mAP was attained using the additional loss which seeks to provide more clear temporal boundaries. ANet1.3 videos are mostly one action, so reducing the temporal scale is a benefit.

We then evaluated the performance on the UA dataset with all augmentation strategies in Table 6. The results in Table 7 are congruent with what would be expected. The mAP approaches supervised UA results, with higher scores than on ANet1.3. Due to the effectiveness of the extractors and the predictable nature of the dataset, even a small amount of labelled data is sufficient for fair results. As the actions are relatively short compared to the video, the temporal scale was made large to ensure that fine temporal transitions are captured and short action sequences are not washed away.

### 3.3. Combined Comparative Study

Our SPOT work from Table 7 can now complete the main comparative study, and the results are shown in Table 8. When compared with a standard benchmark (THUMOS), extractors on UAs did not experience a significant change in frame rate. The same stack and step sizes provided good results on both datasets despite differing mean annotation lengths, since this configuration ensured that nearly all frames were included in multiple clips. All configurations attained significantly higher scores on UAs. Though the UA video’s static background provides minimal context, in a dataset where spatial location is highly predictive of class, a plain background may be beneficial, as it may provide minimal distraction from the key spatial location feature. As shown in Table 8, our approach achieved good results using a variety of pipelines, including those with limited labeled data. Since all instances of UA classes are visually similar, spatially separated, and temporally repetitive, it is easy for the self-supervised tasks to teach spatiotemporal relationships, and few action instances can capture the distribution of each action.

## 4. Conclusions and Future Directions

Through the comparative study of extractors and modifications of SPOT, this paper’s results include the best supervised performance yet on UAs, useful adjustments to SPOT in this application and the first good semi-supervised results on UAs. The experimental results demonstrate that self-supervised learning can be applied to factory applications in settings with uncommon/mixed camera angles in real-time without hand-designed features.

Though all actions are present for the given station, UAs does not include any indication of the correctness of the action. Few authentic examples of dangerous actions may be collectable and that lack of representation restricts UAs’ applicability. While SPOT’s benchmark results suggest good performance on spatially similar classes (and hence the ability to differentiate from subtleties), extending SPOT for anomaly detection is needed and planned.

One of the challenges regarding SPOT’s temporal sensitivity is that each temporal location (clip) is given a class. Hence, the clip size is the finest granularity that the action recognition can handle. Some heads such as ActionFormer return a prediction for each clip for the beginning and ending time of the action to which that clip belongs. Though this would add complexity, it would in theory allow much more accurate predictions.

Ethical vulnerabilities and potential solutions have been addressed herein. All technology related to surveillance could be used punitively; packaging the model in an assistance tool frontend rather than a monitoring system can assist here. Prior to training, the video footage could be intelligently anonymized to hide identifying facial details without interfering with the model’s performance. Each factory should use its own deployed model without data from other factories; not only are different factories likely unrelated, but membership inference attacks may become more prevalent.

As an evolution of simple cropping (Table 4), a fast object detector could be used to localize the hands of the worker, yielding a window of particular interest for usage in an additional model branch. Suitable results may be attainable through a combination of unsupervised and contrastive prototypical learning [46] with the potential to further reduce annotation needs to one instance per class. A non-real-time but powerful model such as VideoMAEv2 [19] could adopt the role of the human-in-the-loop function within a lifelong learning system. It may assist with challenging cases as measured by the entropy and diversity of predictions.

## Figures and Tables

**Table 1:** A review of self-supervised TAL heads.

SSL Head	Description	Limitations	amAP% *
SSP [23]	Mean Teacher model to ensure consistency Sequential perturbations as pretext tasks: – Time Warping: warps input signals using a random flow-field grid – Time Masking: predicts masked features	Perturbations disrespect temporal relationships	38.9
SSTAP [24]	Temporal-aware branch: SSP, but the perturbations maintain temporal relations Separate relation-aware branch for masking	Localization-only implies that errors are propagated forward to the external classifier	40.7
SPOT [25]	Unites localization and classification in parallel	Lacking localization sensitivity, since the clip is the smallest prediction unit	49.9
Ours [26]	SPOT, with modifications: – Losses to encourage hard action and background boundaries – Pretraining–training alternation for earlier task introduction	Further flexibility of localization predictions Lifelong learning system	56.2

* mAP@IoU=0.5 on ANet1.3, 10% labelled.

**Table 2:** UAs statistics compared with large standard TAL benchmarks. UAs is similar in the number of videos and the number of labeled actions to the THUMOS TAL subset. UAs has shorter videos. UAs has a similar number of instances and actions per video, suggesting that classes represented in a UA video are represented once. Only about 15% of the frames in a UA video pertain to an action, suggesting a significant action/background imbalance.

Dataset	#Videos	#Actions	Duration (s)	Instances/Video	Actions/Video	%Action/Video	mAP *
UAs	995	19	71.63	8.16	8.11	15	85%
THUMOS [32]	412	20	213	15.4	1.15	25	71%
ANet1.3 [31]	19,228	200	117	1.15	0.74	65	53.5%

* avg. mAP@IoU=0.5; supervised using I3D and ActionFormer.

**Table 3:** A comparison of feature extractions on UAs. Various architectures and clip sizes were used for the extraction and compared against the extraction time and the downstream performance of the features.

UAs			Clip Size		Time		
Setting	Model	Params	Stack	Step	h:m:s	Real *	amAP **
Vg	VideoMAEv2-giant	1 B	16	16	125:48:53	6.3×	87.69
Vs	VideoMAEv2-small	20 M	16	16	39:08:00	2.0×	82.67
R18	r2plus1d_18_16_kinetics	31.5 M	16	4	6:44:13	0.34×	84.10
R3432-164	r2plus1d_34_32_ig65m_kinetics	31.3 M	16	4	6:44:13	0.44×	82.26
R3432-32	r2plus1d_34_32_ig65m_kinetics	31.3 M	32	32	4:25:13	0.22×	42.81
R348-164	r2plus1d_34_8_ig65m_kinetics	31.3 M	16	4	10:46:45	0.54×	82.78
R348-8	r2plus1d_34_8_ig65m_kinetics	31.3 M	8	8	5:38:31	0.28×	80.14
TSP-R21D-16	r2plus1d_34-tsp_on_thumos14	31.3 M	16	16	1:30:36	0.08×	73.49
TSP-R21D-4	r2plus1d_34-tsp_on_thumos14	31.3 M	16	4	6:46:49	0.34×	84.48
TSP-R21D-1	r2plus1d_34-tsp_on_thumos14	31.3 M	16	1	32:19:55	1.63×	84.89
I3D-164	I3D (rgb)	12.7 M	16	4	10:30:10	0.53×	85.00
I3D-R-164	I3D (rgb + flow)	25.4 M	16	4	30:07:35	1.52×	85.59

* The ratio of the time taken to extract to the total time of the UAs dataset (hh:mm:ss = 19:47:55); **Real** < 1.0× is therefore a “real-time” extractor setting. ** amAP refers to the avg. mAP@IoU=0.1:0.7 using our implemented ActionFormer [17].

**Table 4:** A summary of the effects of the mild crop on the UAs dataset. As the cropped and uncropped versions are both resized to the same shape, there was no significant difference in runtime.

		Uncropped	Cropped		
Model	Side	mAP	Avg. % of Original Size	mAP *	ΔmAP
VideoMAEv2	left	83.64	81	83.89	+0.25
VideoMAEv2	right	89.95	50	90.34	+0.39
R(2+1)D	left	77.24	81	77.63	+0.39
R(2+1)D	right	87.95	50	89.00	+1.05

* amAP@IoU=0.1:0.7 using the ActionFormer head.

**Table 5:** A direct comparison of the classification/localization heads (ActionFormer and TemporalMaxer) on the datasets. The inference time is given for the entire test set.

		ActionFormer		TemporalMaxer	
Extractor	Dataset	amAP	Inference Time	amAP	Inference Time
VideoMAEv2	UA	86.59	34.16 s	87.31	56.85 s
I3D (rgb)	UA	85.00	33.42 s	82.80	32.71 s
I3D (rgb+RAFT)	UA	85.59	34.16 s	84.24	33.89 s
R(2+1)D	UA	84.09	58.58 s	83.23	53.50 s

**Table 6:** An ablation study of the improvements made to SPOT during this project outlined in Section 2.2.2. mAP refers to mAP@IoU=0.5, 10% labelled ANet1.3, for which 49.9% is the result published in the SPOT paper [25].

Contribution	A	B	C	D	E	mAP
Initial implementation						42.377
A: Pretraining-Training Alternation	X					43.433
B. Reconstruction loss for temp. crop	X	X				43.924
C. NLLLoss for Classification	X	X	X			45.931
D. MSE+contrast for Localization	X	X	X	X		47.856
E. Reduced Temporal Scale	X	X	X	X	X	56.216

**Table 7:** Test results for SPOT on the UAs dataset.

Labeled	0.1	0.2	0.3	0.4	0.5	0.6	0.7	0.1:0.7
10%	82.77	81.23	74.89	69.21	65.60	54.24	28.59	65.22
60%	89.12	88.34	85.42	80.05	75.58	65.23	45.85	75.66

**Table 8:** A comprehensive collection of our results. For each combination of (dataset, extractor, head), we present the highest-amAP configuration (as taken from Table 3).

Data		Architecture			Clip Size		Metrics	
Dataset	Label%	Extractor	Flow	Head	Step	Stack	Real *	amAP% **
THUMOS	100	I3D	None	ActionFormer	4	16	0.4×	61.10
THUMOS	100	I3D	RAFT	ActionFormer	4	16	10×	65.27
THUMOS	100	I3D	RAFT	TemporalMaxer	4	16	10×	71.75
THUMOS	100	R(2+1)D	None	ActionFormer	4	16	0.33×	55.18
THUMOS	100	R(2+1)D	RAFT	ActionFormer	4	16	5.0×	62.35
THUMOS	100	R(2+1)D+TSP	None	ActionFormer	4	16	0.34×	60.87
THUMOS	100	R(2+1)D+TSP	None	ActionFormer	1	16	1.63×	61.87
THUMOS	100	VideoMAEv2-g	None	ActionFormer	16	16	6.3×	73.28
THUMOS	100	VideoMAEv2-g	None	TemporalMaxer	16	16	6.3×	74.48
UAs	100	I3D	None	ActionFormer	4	16	0.53×	85.00
UAs	100	I3D	None	TemporalMaxer	4	16	0.53×	82.80
UAs	100	I3D	RAFT	ActionFormer	4	16	1.52×	85.59
UAs	100	I3D	RAFT	TemporalMaxer	4	16	1.52×	84.24
UAs	100	R(2+1)D	None	ActionFormer	4	16	0.34×	84.10
UAs	100	R(2+1)D	None	TemporalMaxer	4	16	0.34×	83.23
UAs	100	R(2+1)D+TSP	None	ActionFormer	4	16	0.34×	84.48
UAs	100	R(2+1)D+TSP	None	ActionFormer	1	16	1.63×	84.89
UAs	100	VideoMAEv2-s	None	ActionFormer	16	16	2.0×	82.67
UAs	100	VideoMAEv2-g	None	ActionFormer	16	16	6.3×	87.69
UAs	100	VideoMAEv2-g	None	TemporalMaxer	16	16	6.3×	86.59
UAs	10	I3D	None	SPOT	4	16	0.53×	65.22
UAs	60	I3D	None	SPOT	4	16	0.53×	75.66

* The ratio of the time taken to extract to the total time of the dataset; **Real** < 1.0× is therefore a “real-time” extractor setting. ** amAP refers to the avg. mAP@IoU=0.1:0.7.

## Data Availability

The factory footage used in this paper is currently unavailable due to privacy and ethical restrictions. However, the THUMOS’14 and ANet1.3 datasets are public benchmarks and can be accessed through the references provided in the paper.

## Abbreviations

The following abbreviations are used in this manuscript:

AP	Average Precision. The ratio of the number of correct predictions to the number of total predictions for a particular class. Ranges from 0 to 1; a greater value is better.
CNN	Convolutional Neural Network
FPS	Frames per second
IoU	Intersection over Union. Given a predicted annotation and a ground-truth annotation, both expressed in terms of start time and end time (one-dimensional “boxes”), IoU is the ratio of the length of the boxes’ intersection (overlap region) to their union (the region where at least one box covers). This measures localization: how well the predicted annotation overlaps with the true action location.
mAP	mean Average Precision. The mean of the (class-wise) APs.
mAP@IoU	mean Average Precision @ Intersection over Union. This is the main metric of TAL. This is the mAP, where “correct” predictions must meet a certain overlap threshold to be considered correct. For example, a mAP@IoU=0.5 requires that a correct prediction has an IoU with a ground truth of at least 0.5. mAP@IoU=0.1:0.7 is the mean of all mAP@IoU=*X*, with X∈{0.1,0.2,…,0.7}.
MDPI	Multidisciplinary Digital Publishing Institute
ML	Machine Learning
MSELoss	Mean Squared Error Loss. The sum of the squared differences between SPOT’s actionness scores at particular temporal locations with the ground truth. This amplifies the effect of large errors.
NLLLoss	Negative Log-Likelihood Loss. For each temporal location, the network will produce logits (converted to log probabilities) for each action class. NLLLoss relates to the predicted probability of the correct class occurring at a given time interval.
PLM	Predicted Localization Mask
RGB	Red, Green, Blue (Color Model)
SOTA	State of the art
SSL	Self-Supervised Learning
TAL	Temporal Action Localization
UAs	Undercover Actions (Dataset)

## Appendix A

We prove in this section that the variance term is no larger than 0.25, so it is safe to subtract it from 0.25 for the purpose of a loss (i.e., no negative losses). This result is then used for the construction of Equation (1).

**Background.** The predicted localization mask (PLM) is a vector $X=[x_{1},\ldots ,x_{T}]$, where all $x_{1}\in \left[0,1\right]$. We have mean $\mu_{X}=\frac{1}{n}\sum_{i=1}^{n}x_{i}$ and variance $\sigma_{X}^{2}=\frac{1}{n}\sum_{i=1}^{n}{\left(x_{i}-\mu_{X}\right)}^{2}$. Substituting $\mu_{X}$ gives $\sigma_{X}=\frac{1}{n}\sum_{i=1}^{n}x_{i}^{2}-\mu_{X}^{2}$, in which the variance is equal to the mean of the squares subtract the square of the mean.

**Lemma** **A1.**
*For a PLM, the variance is maximized when $\forall x_{t}\in PLM,x_{t}\in \left\{0,1\right\}$.*

**Proof of Lemma** **A1.** The sum of squared deviations is

(A1) $$S=n\sigma_{x}^{2}=\sum_{i=1}^{n}{\left(x_{i}-\mu \right)}^{2}=\sum_{i=1}^{n}x_{i}^{2}-n\mu^{2}$$

Let us explore what happens to $\sum_{i=1}^{n}x_{i}^{2}$ and $n\mu^{2}$ when we change some $x_{i^{\prime }}$ to some other $x_{j}$. The change in these is as follows:

(A2) $$\Delta \left(\sum_{i=1}^{n}x_{i}^{2}\right)=x_{j}^{2}-x_{i^{\prime }}^{2}$$

(A3) $$\Delta \left(n\mu^{2}\right)=n\left({\left(\mu +\frac{x_{j}-x_{i^{\prime }}}{n}\right)}^{2}-\mu^{2}\right)=2\mu \left(x_{j}-x_{i^{\prime }}\right)-\frac{{\left(x_{j}-x_{i^{\prime }}\right)}^{2}}{n}$$

We can express the change in *S* as the sum of the changes of its terms:

(A4)
$$\Delta S=\left(x_{j}^{2}-1\right)+\left(2\mu \left(1-x_{j}\right)+\frac{1-x_{j}^{2}}{n}\right)=\left(x_{j}^{2}-1\right)+\left(2\left(\frac{\left(\frac{n}{2}-1\right)+x_{j}}{n}\right)\left(1-x_{j}\right)+\frac{1-x_{j}^{2}}{n}\right)$$

Equation (A4) can be simplified and represented as

(A5) $$\Delta S=\left(1-\frac{1}{n}\right)x_{j}^{2}+2\left(\frac{1}{n}-\mu \right)x_{j}-x_{i}\left(-x_{i}+2\mu -\frac{x_{i}}{j}\right)$$

This is a concave-up parabola with vertex at $x_{j}=\frac{\mu n-x_{i}}{n-1}$ that intersects the $x_{j}$ axis at $x_{i^{\prime }}$ and $\frac{2\mu n-x_{i^{\prime }}\left(n+1\right)}{n-1}$. For some fixed $x_{i^{\prime }}$, $\mu \in [\frac{x_{i}}{n},\frac{n-1+x_{i}}{n-1}]$, which occurs at the cases where $\forall x_{i\neq i^{\prime }}=0$ or $\forall x_{i\neq i^{\prime }}=1$, respectively. Therefore, the vertex, as are $x_{i^{\prime }}$ and $x_{j}$ by definition, are in [0,1]. The parabola will increase monotonically in both directions from the vertex, so the most positive ΔS will occur at the endpoints of the domain of ΔS, where $x_{j}\in \left\{0,1\right\}$. Note that fully maximizing *S* requires the exchange from $x_{i^{\prime }}$ to $x_{j}$ to occur for all $x_{i}\in X$. Consider a case $X=[x_{1},\ldots ,x_{T-1},x_{i^{\prime }}\in \left\{0,1\right\}]$, where $\forall x_{j},\Delta S\leq 0$, such as $X=[0.5,0.5,0.5,1=x_{i^{\prime }}]$. Clearly $\exists x_{i}\notin \left\{0,1\right\}$, but this simply means that $x_{i^{\prime }}$ cannot be further maximized (it should stay at an endpoint) and we should choose a different $x_{j}$. Therefore, we maximize *S* by sending all $x_{i}$ to 0 or 1. □

**Theorem** **A1.**
*$\sigma_{PLM}^{2}\in \left[0,0.25\right]$.*

**Proof of Theorem** **A1.** By Lemma A1, $\forall x_{i}\in X,x_{i}\in \left\{0,1\right\}$. Assume that there are 1≤k≤n predictions/elements that are 1, and the remaining n−k are 0. Therefore, the mean of *X* is $\mu =\frac{k}{n}$. Based on this, the variance of the PLM *X* is

(A6) $$\sigma_{X}^{2}=\frac{1}{n}\sum_{i=1}^{n}{\left(x_{i}-\mu \right)}^{2}=\frac{1}{n}\left[k{\left(\frac{n-k}{n}\right)}^{2}+\left(n-k\right){\left(\frac{k}{n}\right)}^{2}\right]=\frac{k(n-k)}{n^{2}}$$

The derivative of $\sigma_{X}^{2}$ with respect to *k* is $\frac{n-2k}{n^{2}}$, which is maximized at $k=\frac{n}{2}$. Therefore, half of the elements should be 0, and the rest should be 1. Substituting, this means that $\sigma_{X}^{2}=\frac{k(n-k)}{n^{2}}=1/4=0.25$ when maximized. And, since the variance is a sum of squared terms, it must always be non-negative. □

## Appendix B

These are the equations that compute the action/background-wise prediction means. $x_{t}\in \left[0,1\right]$ is the predicted confidence score that there is an action at clip $x_{t}$. $y_{t}\in \left\{0,1\right\}$ is the actionness of the ground truth, $y_{t}=1$ if the clip at time *t* is part of an annotation. By construction, Equation (A7) is (approximately) the average confidence score of the scores that are located at a ground-truth action, Equation (A8) does the same for the background (1−y), providing the correct filtering.

(A7) $${\bar{x}}_{action}=\frac{1}{\sum_{t}y_{t}+1}\cdot \sum_{t}x_{t}y_{t}$$

(A8) $${\bar{x}}_{background}=\frac{1}{\sum_{t}\left(1-y_{t}\right)+1}\cdot \sum_{t}x_{t}\left(1-y_{t}\right)$$

## Appendix C

This Appendix outlines the environment details used in these experiments. Table A1 lists the software and code repositories used. Table A2 shows the hyperparameters that are kept constant for all supervised head experiments.

**Table A1:** A brief summary of the software tools used in the comparative study.

	Models							
	VideoMAEv2	I3D	RAFT	R(2+1)D	TSP	ActionFormer	TemporalMaxer	SPOT
OS	Ubuntu 20.04							
GPUs	1x NVIDIA A100 80GB PCIe							
CUDA	12.2							
Python	3.8.10	3.8.5			3.10.13	3.8	3.9	3.8.10
PyTorch	1.12.1+cu113	1.7.1=py3.8_cuda11.0.221_cudnn8.0.5_0			1.11.0	1.11.0+cu113	1.12.1	1.11.0+cu113
Original Code	[19]	[47]			[33]	[35]	[18]	[25]
(Forked) Codebase	[48]	[49]			[50]	[17]	[51]	[26]

**Table A2:** The hyperparameter settings for the ActionFormer and TemporalMaxer heads.

Hyperparameter	Value
Training Epochs	ActionFormer: 30, TemporalMaxer: 60
Learning Rate	0.0001
Momentum	0.9
Optimizer	AdamW [52]
Warmup Epochs	5
Weight Decay	0.05
Kernel Size	3
Step Size	4
Stack Size	16

## Appendix D

This appendix provides background details about the hyperparameters and experiments used for the regression analysis in Table A3. In Table A4, the parameters are presented, along with the default value of the parameter as used by SPOT for ANet1.3 and the range of values used in the parameter sweep with selection justified herein. The full table of experiments during the sweep used as data for the linear regression is provided in Table A5. All of these results were used to empirically determine the parameters of the contributions from Table 6. The following is a brief explanation of the hyperparameters used:

**EmbeddingHead:** The number of attention heads in the transformer used for embedding. In addition to the default of four heads, a single head is studied to inspect if fewer attention heads is sufficient and reduces the overfitting on UAs.

**TrainingStep:** Every **TrainingStep** epoch, the learning rate is multiplied by **Gamma**. Lower values may provide faster convergence, but it may be suboptimal depending on local minima.

**Gamma:** The γ for the StepLR scheduler. γ=0.8 will take many more epochs to converge.

**LossBalance:** A weighing factor for the branch losses. The combined branch loss is given as **LossBalance** · TopLoss + (1 − **LossBalance**) · BottomLoss. The TopLoss refers to the top (classification) branch, and the BottomLoss refers to the bottom (localization) branch.

**Lambda2:** A weighing factor for the F-Loss on the bottom (localization) branch. The loss is given as $F=\lambda_{2}BCELoss+\left(1-\lambda_{2}\right)DICELoss$. Note that $\lambda_{2}\approx 0\Rightarrow F\approx DICELoss$ will prioritize the segment level rather than the frame level, and the opposite is true when $\lambda_{2}$ is large.

**Table A3:** Linear regression for SPOT on ANet1.3. Analyzes how select hyperparameters correlate with the mAP and time to train. It is computed from the experimental data in Table A5.

	mAP@IoU=0.5			Training Time		
Coefficient	Estimate	*p*-Value	Significance	Estimate	*p*-Value	Significance
EmbeddingHead	−0.35	$2.94\times 10^{-7}$	***	−6.17	0.73	
TrainingStep	0.015	0.67		20.18	0.06	.
Gamma	−0.17	0.57		−3.05	0.97	
LossBalance	−0.87	$3.36\times 10^{-3}$	**	129	0.12	
LambdaTwo	0.42	0.11		−16.0	0.84	

Significance codes: 0 ‘***’ 0.001 ‘**’ 0.01 ‘.’ 0.1 ‘ ’ 1.

Interestingly, EmbeddingHead is both highly significant and negative, suggesting that fewer heads may be better. Despite the best efforts of self-supervised pretraining, with only 10% of the dataset labeled, overfitting is always a prime suspect. However, the margins are fine; all rows in the table fall within 4.5 mAP, so there is no catastrophic overfitting due to the extra heads. The other term that is significant at (standard) significance level α=0.05 is the LossBalance. This term is also negative, indicating that it is preferable to up-weigh the bottom (localization) branch. In SPOT’s setup, the classification branch is more complex, with two 1D convolutions instead of one. It is possible that the more complex top branch could overfit if it is prioritized too heavily. In general, localization is considered more difficult than classification, especially if it is temporal localization. Additionally, 0.5 is a non-trivial IoU, so poor localization will be punished. None of these explanations are mutually exclusive. As no early stopping mechanism is employed, the training time is mostly unaffected by the factors.

**Table A4:** The ranges of hyperparameters used in Table A5.

Parameter	Range	Default
EmbeddingHead	{1, 4}	4
TrainingStep	{5, 10}	10
Gamma	{0.2, 0.8}	0.2
LossBalance	{0.1, 0.5, 0.9}	0.5
Lambda2	{0.1, 0.4, 0.9}	0.4

**Table A5:** A parameter sweep of various hyperparameters for ANet1.3, 10% labeled. These rows were analyzed using linear regression in Table A3.

EmbeddingHead	TrainingStep	Gamma	LossBalance	Lambda2	mAP *	Time **
1	10	0.2	0.1	0.1	46.293	2282.234
1	10	0.2	0.1	0.4	46.642	2270.906
1	10	0.2	0.1	0.9	45.385	2138.472
1	10	0.2	0.5	0.1	44.234	2681.148
1	5	0.2	0.1	0.4	46.642	2148.239
1	5	0.2	0.1	0.9	45.385	2178.62
1	5	0.2	0.5	0.1	44.073	2143.392
1	5	0.2	0.5	0.4	45.78	2168.776
1	5	0.2	0.5	0.9	44.545	2160.215
1	5	0.2	0.9	0.1	44.271	2130.248
1	5	0.2	0.9	0.9	44.814	2293.03
1	5	0.8	0.1	0.9	45.345	2132.818
1	5	0.8	0.5	0.1	43.406	2112.199
1	5	0.8	0.5	0.4	45.348	2147.939
1	5	0.8	0.5	0.9	44.963	2141.788
1	5	0.8	0.9	0.1	43.944	2185.66
1	5	0.8	0.9	0.4	44.211	2170.468
1	5	0.8	0.9	0.9	45.023	2223.197
4	10	0.2	0.1	0.1	42.404	2282.896
4	10	0.2	0.1	0.4	44.687	2257.02
4	10	0.2	0.1	0.9	44.816	2244.643
4	10	0.2	0.5	0.1	44.071	2246.196
4	5	0.2	0.1	0.1	42.404	2238.389
4	5	0.2	0.1	0.4	44.729	2270.642
4	5	0.2	0.1	0.9	44.816	2277.225
4	5	0.2	0.5	0.1	43.953	2280.079
4	5	0.2	0.5	0.4	44.027	2280.583
4	5	0.2	0.5	0.9	43.663	2293.313
4	5	0.2	0.9	0.1	44.447	2236.358
4	5	0.2	0.9	0.9	43.807	2355.798
4	5	0.8	0.1	0.1	42.404	2204.206
4	5	0.8	0.1	0.4	44.71	2247.803
4	5	0.8	0.1	0.9	44.816	2222.575
4	5	0.8	0.5	0.1	44.053	2184.01
4	5	0.8	0.5	0.4	43.904	2269.515
4	5	0.8	0.5	0.9	43.678	2253.94
4	5	0.8	0.9	0.1	44.333	2250.151
4	5	0.8	0.9	0.4	43.421	2292.874
4	5	0.8	0.9	0.9	44.109	2235.912
1	10	0.2	0.5	0.4	45.364	3169.779
1	10	0.2	0.5	0.9	44.689	2142.15
1	10	0.2	0.9	0.1	44.175	2130.564
1	10	0.2	0.9	0.4	44.284	2180.587
1	10	0.2	0.9	0.9	44.836	2163.776
1	10	0.8	0.1	0.1	46.31	2159.365
1	10	0.8	0.1	0.4	46.642	2156.34
1	10	0.8	0.1	0.9	45.338	2150.775
1	10	0.8	0.5	0.1	43.874	2148.413
1	10	0.8	0.5	0.4	45.233	2135.986
1	10	0.8	0.5	0.9	44.346	2133.672
1	10	0.8	0.9	0.1	44.266	3163.667
1	10	0.8	0.9	0.9	44.693	3217.362
4	10	0.2	0.5	0.4	44.043	2254.155
4	10	0.2	0.5	0.9	43.672	2254.956
4	10	0.2	0.9	0.1	44.309	2282.938
4	10	0.2	0.9	0.4	43.77	2244.922
4	10	0.2	0.9	0.9	43.526	2250.859
4	10	0.8	0.1	0.1	42.404	2271.158
4	10	0.8	0.1	0.4	44.687	2276.477
4	10	0.8	0.1	0.9	44.817	2250.44
4	10	0.8	0.5	0.1	43.983	2246.682
4	10	0.8	0.5	0.4	44.003	2269.359
4	10	0.8	0.5	0.9	43.651	2252.148
4	10	0.8	0.9	0.1	44.276	2264.298
4	10	0.8	0.9	0.4	43.597	2247.971
4	10	0.8	0.9	0.9	43.53	2237.524

* mAP@IoU=0.5, ANet, 10% labelled, using I3D features from [25]. ** Time (seconds) to complete the entire training phase.
